# Distribution Patterns in the Native Vascular Flora of Iceland

**DOI:** 10.1371/journal.pone.0102916

**Published:** 2014-07-18

**Authors:** Pawel Wasowicz, Andrzej Pasierbiński, Ewa Maria Przedpelska-Wasowicz, Hörður Kristinsson

**Affiliations:** 1 Icelandic Institute of Natural History, Borgir við Norðurslóð, Akureyri, Iceland; 2 Department of Plant Systematics, Faculty of Biology and Environmental Protection, University of Silesia, Katowice, Poland; 3 Institute of Botany, University of Warsaw, Warszawa, Poland; Royal Botanic Gardens, Kew, United Kingdom

## Abstract

The aim of our study was to reveal biogeographical patterns in the native vascular flora of Iceland and to define ecological factors responsible for these patterns. We analysed dataset of more than 500,000 records containing information on the occurrence of vascular plants. Analysis of ecological factors included climatic (derived from WORLDCLIM data), topographic (calculated from digital elevation model) and geological (bedrock characteristics) variables. Spherical k-means clustering and principal component analysis were used to detect biogeographical patterns and to study the factors responsible for them. We defined 10 biotic elements exhibiting different biogeographical patterns. We showed that climatic (temperature-related) and topographic variables were the most important factors contributing to the spatial patterns within the Icelandic vascular flora and that these patterns are almost completely independent of edaphic factors (bedrock type). Our study is the first one to analyse the biogeographical differentiation of the native vascular flora of Iceland.

## Introduction

The detection of patterns in the distribution of organisms is one of the most important tasks in biogeography [1] and a starting point for all biogeographical analyses. When taxa with similar distribution are grouped together (forming clusters that can be called “biotic elements”) complex reality is simplified and reduced to the smaller number of components. This approach allows us to generate hypotheses about factors that contribute to the emergence of biogeographical patterns and, finally, to cross the bridge between the hypothesis-generating phase and the hypothesis-testing phase of biogeographical research [2].

The task of detection of biotic elements is limited by both species distribution data (amount, quality and availability) and methodology. Availability of species distribution data (particularly in electronic form) has increased rapidly over the last decades, especially with the advent of Internet-based infrastructure projects like GBIF (http://www.gbif.org) or LifeWatch (http://www.lifewatch.eu). However, the amount and quality (both taxonomic reliability and spatial resolution) of the data may still be serious limiting factors, especially for studies carried out at a regional scale.

Methods for classifying species into biotic elements with similar distribution patterns remain the main challenges in this field of research. Initially, such classifications were made by eye and were based completely on expert judgment (e.g. [3], [4]). With the advent of increased interest in numerical classification, clustering of multivariate species data became very popular in biogeographical studies (e.g. [5], [6]). This approach facilitated more objective classifications than those generated previously. There are different methods and techniques of numerical classification available to assist the biogeographer, including canonical correspondence analysis [7], detrended correspondence analysis [8] and two way indicator species analysis [9]. Most techniques currently in use classify the samples first and then the species [10], but this can lead to the underestimation of relatively subtle (but important) differences between species occurrence patterns [10]. Recently, a new approach to the analysis of multivariate species occurrence data was described by Hill et al. [10] and was found to be effective in overcoming some of the limitations described above [11], [12].

There are 438 species in the vascular flora of Iceland [13]. Flowering plants account for 92% of the total number of species and ferns for about 8%. Boreal species (boreo-temperate, boreal-montane and wide-boreal) are very abundant and account for 46% of the native flora, while Arctic and Boreo-Arctic species account for 37% of the total. Temperate species are amongst the least numerous and account just for 18% of the native flora. The flora of Iceland has unique features, and differs from both Greenland and Scandinavia in having an Atlantic European element that is more abundant than in any other part of the Arctic and Subarctic [14]. Extensive bird migration and the presence of unique ecological niches (geothermal areas) also contribute to specific features of the flora. Most taxa in the Icelandic flora are of European origin, which is surprising as Iceland is situated much closer to Greenland than to any part of Europe. Iceland was extensively glaciated during the Pleistocene and its entire flora is now considered to be of postglacial origin [14]. Due to its young age, the flora lacks endemics at the species level, but numerous taxa exhibit subtle morphological differences when compared with accessions from mainland Europe. The northernmost peninsulas of Iceland are considered as a part of the Arctic according to the Panarctic Flora Checklist and, with Jan Mayen, are treated as one floristic region [14]. The rest of its territory is considered to be part of the Subarctic [15].

Data on the distribution of Icelandic vascular plants have been systematically collected since the late 19th century [16]–[18]. Highly reliable, but general distribution maps were published in *The Guide to Flowering Plants and Ferns of Iceland* [19]. These maps, as well as our present study, are based on the information from a database that was founded in 1970 and is now housed in the Icelandic Institute of Natural History (data are publicly available through GBIF). The database provides access to all available data on the distribution of vascular plants in Iceland: published and unpublished, field records as well as herbarium specimens kept in Icelandic herbaria. It provides an access to high-resolution spatial data that have been taxonomically revised, thus creating an excellent opportunity to carry out biogeographical research.

With the passage of time, large data resources have accumulated, but there are still no studies accessible to the international research community and offering a synthesis of phytogeographical data from Iceland. The only papers published hitherto have a relatively narrow scope and are mainly in Icelandic [20]–[23]. With the exception of the recently published study on alien plant taxa [24], phytogeographical studies on Icelandic vascular flora remain neglected, and this is reflected in their absence from the international scientific literature.

Recent assessments [15] have shown that basic knowledge on the vast majority of Arctic biodiversity is limited. Even though the distribution of vascular plants seems to be one of the better documented features, phytogeographical studies focused on Arctic and Subarctic areas are still poorly represented in the literature, compared with the abundance of studies from lower latitudes. This fact has its roots in the low accessibility of Arctic and Subarctic areas, harsh climatic conditions, low population density and consequently scarcity of data. Recently, huge efforts have been undertaken to delineate borders of the Arctic and Subarctic territory on the basis of phytogeographical and ecological data [14], [25], [26] and to define bioclimatic subzones of the Arctic territory [26]. In this context, Iceland can be considered as one of the very few regions located in the Arctic and Subarctic with a wealth of high-resolution floristic data. This fact creates excellent and unique opportunities for phytogeographical studies in general, and for studies based on numerical classifications in particular.

Taking into account the huge changes in the distribution of arctic vegetation that are predicted to take place in forthcoming years [27] it seemed to us important to design a study that would document present phytogeographical patterns and thus facilitate the tracking of future range shifts caused by climate change.

In the present study we employed both accurate and reliable data sources as well as the latest methodology to define biotic elements within the vascular flora of Iceland. Using GIS tools we also tested hypotheses on the factors that might be responsible for the presence of these elements in the native vascular flora of Iceland.

We aimed to achieve the following aims:

1. To reveal biogeographical patterns in the native vascular flora of Iceland.
2. To investigate potential ecological factors responsible for these patterns by testing the hypotheses that spatial patterns in the native flora of Iceland are controlled by: (i) climatic, (ii) topographic and (iii) geological factors.

## Materials and Methods

### Data sources

Distribution data were obtained from the database of the Icelandic Institute of Natural History. Analysis was restricted to native or doubtfully native species. Alien taxa (both established and casual) [24] were excluded from analyses. Records of infraspecific taxa were included with those of the appropriate species and their nomenclature follows Kristinsson [13]. Records of native species in cultivation or spreading from cultivation were excluded from the dataset. We included all the records irrespective of date. The whole area of Iceland was divided into hectads (10×10 km square) and each species record was assigned to appropriate hectad. The distribution of 438 species in 1,108 hectads was analysed. In total 517,663 records were retrieved from the database and used during the present study. All the records are publicly available through the GBIF database (see Text S2 for details).

In order to assess the biogeographical affinities of species within each cluster we assigned each species to wide biogeographical elements: arctic-montane, boreo-arctic montane, wide-boreal, boreal-montane, boreo-temperate, wide-temperate, temperate, southern-temperate, mediterranean-atlantic, mediterranean. This assignment was based on published reference data [28], [29]. Biogeographical affinities were expressed as the percentage of the total number of species within a given cluster and visualised on bar plots. Information on the life form (chamaephyte, hemicryptophyte, hydrophyte, nanophanerophyte, phanerophyte, therophyte) [30] was also gathered for each taxon. IUCN categories were assigned according to Icelandic Red List of Plants [31].

Climatic data were downloaded from WORLDCLIM database–worldclim.org [32]. The data are interpolations of observed data, representative of 1950–2000. A set of 19 bioclimatic variables in 30″ resolution was created from input climatic data (WORLDCLIM database) using DIVA GIS 7.5 [33] downloaded from http://www.diva-gis.org/download. Research on topographic variables was based on the digital elevation model for Iceland (20 m per pixel) downloaded from http://gatt.lmi.is. Analysis of edaphic variables was based on the data extracted from the geological map of Iceland 1:600,000 downloaded from http://gatt.lmi.is. Geological data described the bedrock type (five major types were recognised: lava, hyaloclastite, extrusive rock, intrusions, sands), its pH (acidic rocks vs. basic or intermediate rocks) and age (eight age classes were recognised: holocene sediments; postglacial, historic, younger than AD 871; postglacial, prehistoric, older than AD 871; Tertiary and Pleistocene, older than 11000 years; Upper Pleistocene, younger than 0.8 m.y.; Upper Pliocene and Lower Pleistocene, 0.8–3.3 m.y.; Upper Tertiary, older than 3.3 m.y.; Indefinite).

### Data analysis

#### Spherical k-means clustering

Occurrence data were analysed using SPHERIKM [10] downloaded from (http://www.brc.ac.uk/downloads/Spherikm_public_version.zip). This computational approach minimizes the within-group dispersion of k clusters on the surface of a sphere and weights each species by a fixed power *p* of its frequency. It means that the more common the species is, the greater it is weighted. The software identifies ‘key species’ and uses them to initiate the clustering process (so called ‘cluster seeds’). Names of the clusters in the present paper were given after ‘key species’ of each cluster. SPHERIKM also allows one to calculate how well each species fits into the cluster. This is measured by S (cosine of the angle between the species and its cluster centroid). In the present study we used the perpendicular spherical k-means option (PSKM), with weights W = 0.5. Other weights options (W = 0.0 and W = 1.0) were also tested (see discussion). Apart from the number of clusters and weights other parameters were set to default. We analysed a broad range of different k values from 2 to 70. The statistically optimal number of clusters was assessed using quasi-Akaike information criterion [10].

#### Environmental variables

Initially, we carried out a correlation analysis (Pearson correlation coefficient) using R statistical software, ver. 3.0.1 [34] to eliminate bioclimatic variables with Pearson’s r higher than 0.8. This approach enabled us to decrease the rate of redundancy in the data. Finally, 7 out of 19 bioclimatic variables were included in the analysis (Table 1).

**Table 1:** Environmental (bioclimatic and topoclimatic) variables used in the present study.

Variable code	Variable name	unit	Description	Source
BIO1	Annual mean temperature	°C	Interpolation of observed data 1950–2000	WORLDCLIM database
BIO2	Mean diurnal temperature range	°C	t_max_–t_min_, monthly averages	calculated from WORLDCLIM data
BIO3	Isothermality[1]	-	Annual mean temperature/temperatureannual range	calculated from WORLDCLIM data
BIO4	Temperature seasonality	-	Coefficient of variation calculated frommonthly temperature means	calculated from WORLDCLIM data
BIO9	Mean temperature of driest quarter	°C	Mean temperature calculated for thequarter with lowest precipitation	calculated from WORLDCLIM data
BIO12	Annual Precipitation	mm	Total sum of precipitationcalculated from monthly sums	calculated from WORLDCLIM data
BIO15	Precipitation Seasonality	-	Coefficient of variation calculated frommonthly precipitation data	calculated from WORLDCLIM data
ELEV	Elevation	m	Elevation above sea level	http://gatt.lmi.is
TRI	Terrain Ruggedness Index	-	Quantifies topographicheterogeneity	calculated from digital elevation model according to Riley et al. [55]
WI	SAGA Wetness Index	-	Identifies areas with high waterretention potential.	calculated from digital elevation model according to Böhner et al. [57]
DI	Duration of Insolation	h	Total time of potential insolationcalculated for the period betweenMay 1st and September 30th.	calculated from digital elevation model according to Wilson & Gallant [40], Böhner & Antonić [38]
TI	Total Insolation	kWh/m^2^	Total incoming solar radiation calculatedfor the period betweenMay 1st and September 30th.	calculated from digital elevation model according to Wilson & Gallant [40], Böhner & Antonić [38]
MRVBF	Multiresolution Indexof Valley Bottom Flatness	-	Identifies areas that are flat and locallylow. Takes value of less than 0.5 in areasthat are not valley floors (ridges, hilltops, hillslopes)and larger values for progressively flatterand broader areas (valley bottoms).	calculated from digital elevation model according to Gallant & Dowling [56]

[1] The variable called isothermality represents temperature evenness over the course of year (e.g. areas with isothermality value of 100 represent sites where diurnal temperature range equals to the annual temperature range, whereas areas with isothermality value of 50 represent sites where diurnal temperature range is equal to half of the annual temperature range).

A digital elevation model was used to calculate five terrain parameters (Table 1) using SAGA GIS, ver. 2.0.8 (http://www.saga-gis.uni-goetingen.de) and to obtain elevation data for plant localities examined during the present study. All the vector and raster data were projected into the ISN93 coordinate system and values for all the environmental variables were assigned to each analysed locality. After eliminating records with missing data, we obtained a database containing information on the environmental variables for 476,574 localities.

Principal component analysis (PCA) was carried out on the matrix of correlations calculated from the data matrix containing median values of environmental and topographic variables calculated for each cluster. The aim of the PCA was to investigate relations between species clusters (obtained as a result of spherical k-means clustering) and to determine the environmental variables responsible for the differentiation between clusters. Two separate PCA analyses were conducted, one on the climatic and one on the topographic data matrix, in order to maximise the percent of the total variance explained by the first two principal components. Calculations were done using Statistica 12 (Statsoft Inc.).

We tested whether species clusters differ in terms of the frequency of categorical variables describing bedrock-related characteristics. A value for each categorical variable was assigned to each analysed locality. We compared class frequencies in each species cluster to the frequencies obtained by pooling all the data. Statistical significance of the observed differences was tested by the Chi-square test conducted using Statistica 12 (Statsoft Inc.).

## Results

Akaike information criterion (quasi-AIC) [10] suggested that the 438 species were best divided into 45 clusters (Figure S1, Figure S2), which is too many to represent a succinct and meaningful overview. Taking this result into account, and after examining a number of possibilities, we decided to select the number of clusters arbitrarily (see more details in discussion).

We divided the native Icelandic flora into 10 clusters (floristic elements). This choice displays the range of variation well and the number of clusters is not too large to assimilate. The number of component species in each group ranged from 25 to 80 species.

The first three clusters (*Bistorta vivipara*, *Anthoxanthum odoratum* and *Rhinanthus minor*) form a sequence of species with decreasingly ubiquitous ranges.

### *Bistorta vivipara* cluster

**Figure 1:**
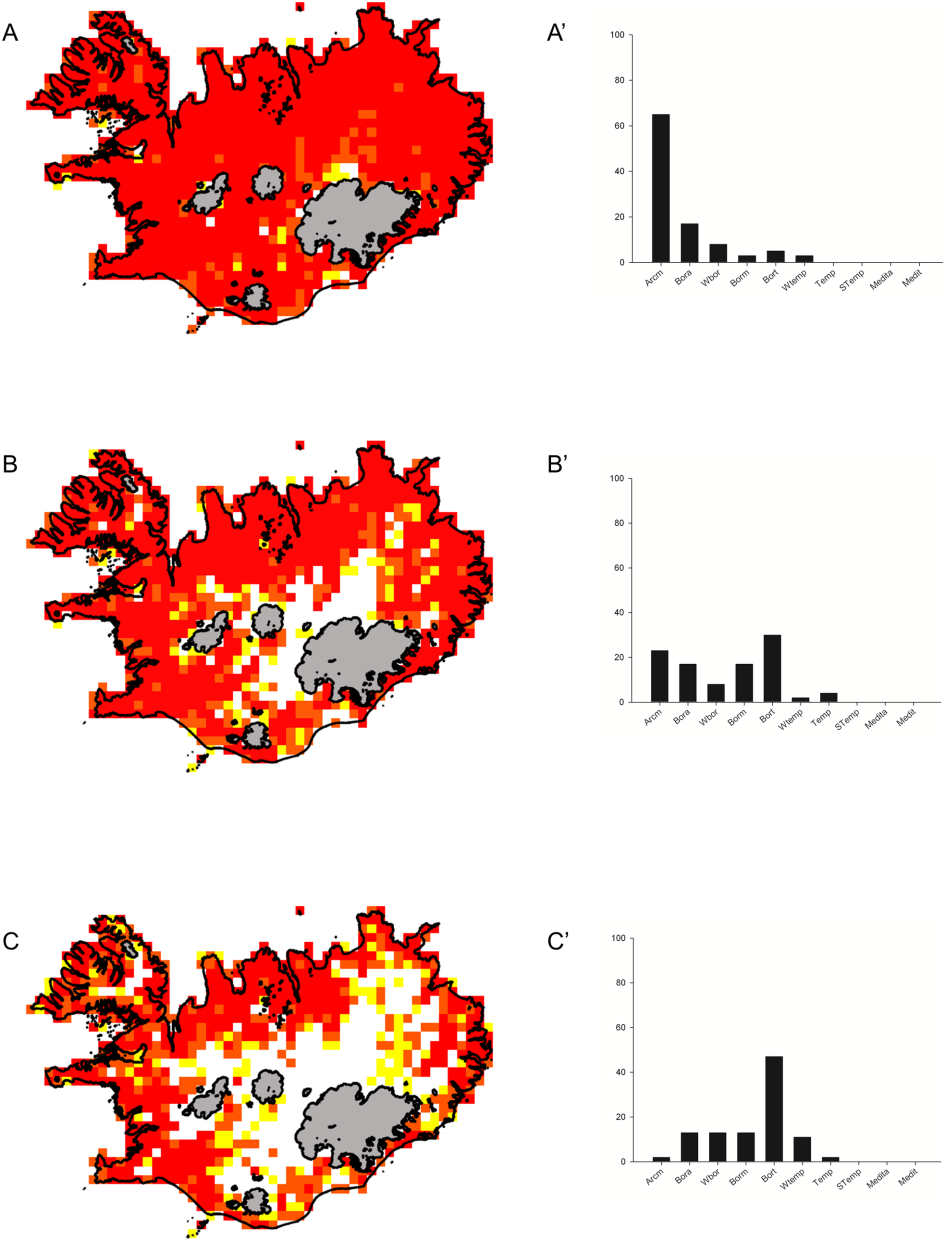
Distribution and biogeographical affinities of species clusters. Maps of species distribution in the clusters: *Bistorta vivipara* (A), *Anthoxanthum odoratum* (B), *Rhinanthus minor* (maps) and their biogeographical affinities (diagrams A’, B’, C’). 10×10 km hectads were marked with colours depending on the percentage of species from the respective cluster occurring in the hectad: 15–25%-yellow, 25–50%-orange, >50%-red. The bars on the biogeographic plots indicate percentage of species in the cluster belonging to major biome categories: Arcm–arctic montane, Bora–boreo-arctic, Wbor–wide boreal, Borm–boreal-montane, Bort–boreo-temperate, Wtemp–wide-temperate, Temp–temperate, Stemp–southern-temperate, Medita–mediterranean-atlantic, Medi–mediterranean. Main glaciers are shaded gray.

The *Bistorta vivipara* cluster has the most widespread distribution (Figure 1A) and it consists of 65 species occurring throughout Iceland. All the species in this cluster fit the cluster patterns well (the lowest S value was 0.69 and 69% of the species have S value≥0.9). This cluster is the only one that covers virtually the whole investigated area including the harsh environments of Central Highlands, the areas bordering glaciers, and the highest mountain ranges. The distribution of the component species is rather continuous and their frequency in almost all analysed hectads was about 80% (50 species were recorded in each hectad on average). Although the species have different major biome ranges (from Arctic to Wide-temperate), Arctic species are dominant and account for 65% of the total number of species in this cluster (Figure 1A’). Boreo-arctic species account for 17%, while other groups (Boreal-montane, Boreo-temperate and Wide-temperate species) account for 19% in total. There are no threatened species in this cluster (Table 2). Hemicryptophytes and chamaephytes are the dominant life forms (Table 2).

**Table 2:** Number of species, number of threatened species according to IUCN criteria and percentage of different life forms in each plant cluster.

Cluster name	No. spp.	IUCNspecies no. (%)	% G	% Ch	% He	% Hy	% Na	% Ph	% Th
*Bistorta vivipara*	65	0 (0)	4.6	27.7	61.5	0	3.1	0	3.1
*Anthoxanthum odoratum*	53	0 (0)	11.3	18.9	56.6	3.8	1.9	3.8	3.8
*Rhinanthus minor*	47	0 (0)	2.1	4.3	59.6	19.1	0	0	14.9
*Luzula arcuata*	25	0 (0)	0	12.0	88.0	0	0	0	0
*Carex rupestris*	26	5 (19)	3.8	11.5	69.2	0	0	0	15.4
*Nardus stricta*	51	8 (15)	19.5	17.1	53.7	2.4	0	2.4	4.9
*Saxifraga aizoides*	25	6 (24)	9.5	19.0	61.9	0	0	9.5	0
*Puccinellia maritima*	30	6 (20)	3.3	3.3	60.0	16.7	0	0	16.7
*Potamogeton alpinus*	38	7 (18)	2.6	2.6	23.7	63.2	0	0	7.9
*Rumex longifolius*	80	14 (17)	8.3	4.2	62.5	6.9	0	0	18.1
Total	440	46 (10)	6.7	12.5	58.8	11.0	0.7	1.2	9.1

G-geophytes, Ch-chamaephytes, He-hemicryptophytes, Hy-hydrophytes, Na-nanophanerophytes, Ph-phanerophytes, Th-therophytes. Percentages in IUCN species column reflect the proportion of threatened species in each cluster.

### *Anthoxanthum odoratum* cluster

The *Anthoxanthum odoratum* cluster comprises 53 species that fit the cluster pattern well (the lowest S value was 0.61 and 39% of the species have S value≥0.9). The *Anthoxanthum odoratum* cluster is still quite widespread (Figure 1B), but clearly species included here do not occur in the central part of the Central Highlands. They are, however, very common in other regions. Species included in this cluster are characterised by wide variation of major biome ranges from arctic to temperate. Boreo-temperate species are most common here and account for 30% of the total number of taxa in the cluster. Arctic species account for 23% while boreal species account for 72% in total. Other groups (wide-temperate and temperate species) account for 6% in total (Figure 1B’). There are no threatened species in this cluster (Table 2). All the life forms are represented in this cluster, but hemicryptophytes, chamaephytes and geophytes are the most common (Table 2).

### *Rhinanthus minor* cluster

The *Rhinanthus minor* cluster comprises 47 species, reasonably well fitted to the cluster pattern (the lowest S value was 0.43 and 57% of the species have S value≥0.8). These are mostly lowland species only rarely extend into the Central Highlands (Figure 1C). Arctic and temperate species are very rare (with only 2% share each), whereas the core of this cluster is formed by Boreo-temperate species (47%). Boreo-arctic, wide-boreal, boreal-montane and wide-temperate species accounting for about 10% each (Figure 1C’). There are no threatened species in this cluster (Table 2). Hemicryptophytes, hydrophytes and therophytes are most common within this group (Table 2).

### *Luzula arcuata* cluster

**Figure 2:**
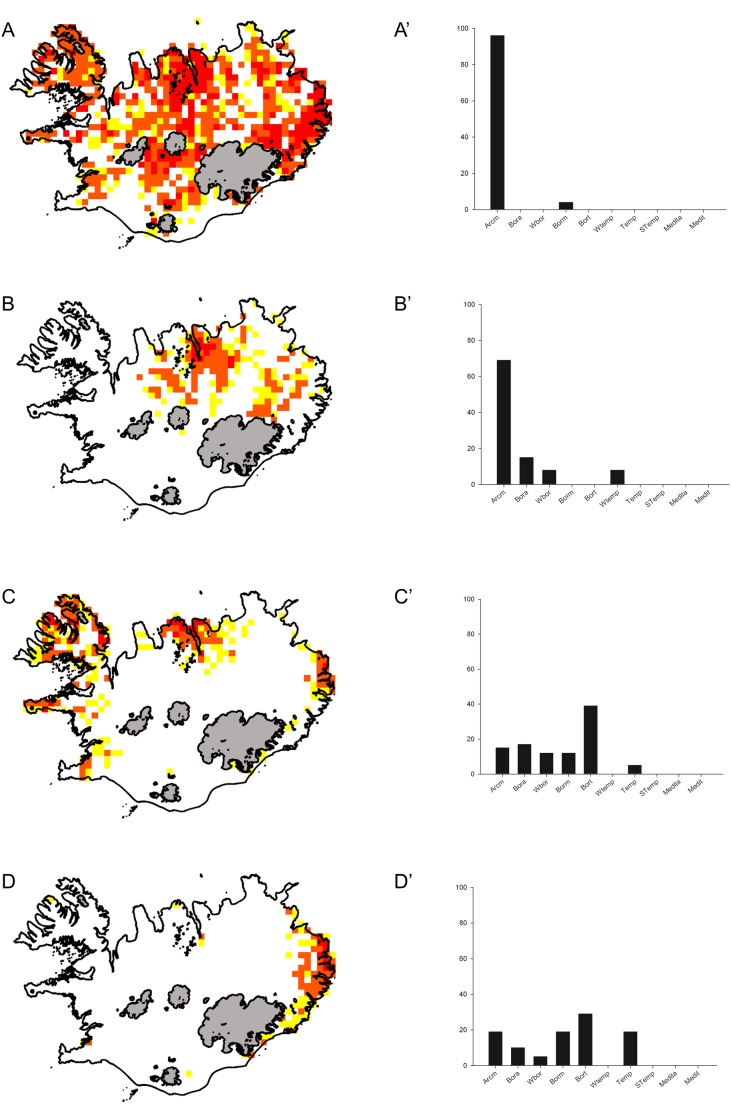
Distribution and biogeographical affinities of species clusters. Maps of species distribution in the clusters: *Luzula arcuata* (A), *Carex rupestris* (B), *Nardus stricta* (C), *Saxifraga aizoides* (D) (maps) and their biogeographical affinities (diagrams A’, B’, C’, D’). For explanations see Figure 1.

This cluster consists of 25 species differing in the degree to which they fit the cluster pattern (the lowest S value was just 0.2, there was no species with S≥0.9, but 76% had the S value≥0.5). Species from this cluster are distributed across Iceland (Figure 2A), but clear hotspots occur in the central part of the northern coast, in the Central Highlands and in the eastern part of the country. Western Fjords (the large peninsula in the north-western part of the country) is also among the regions with a relatively high abundance of species from *Luzula arcuata* cluster. Their occurrence in the south and south-western part of the island seems to be limited. Unlike other clusters, this one is almost completely dominated by arctic species (96%), Boreal-montane species account for the remaining 4% (Figure 2A’). The presence of threatened species was not recorded (Table 2). There are just two life forms represented here: hemicryptophytes and chamaephytes (Table 2).

### *Carex rupestris* cluster

Twenty-six species were assigned to this cluster. Their occurrence is clearly limited to the area extending from the central part of the northern coast, through highlands to the eastern part of the island. It is clear that species from this cluster are almost completely absent from the southern and western parts of the country (Figure 2B). Species assigned to this cluster vary widely in terms of their fit to the cluster pattern (the lowest S value was 0.17, 45% of the species had S value≥0.5, 11% had S value≥0.7,). This cluster is also dominated by arctic species (69%). Boreo-arctic species are the second in terms of abundance, accounting for 15%. Wide-boreal and wide-temperate species account for 8% each (Figure 2B’). The presence of five threatened species was recorded in this cluster. Hemicryptophytes, therophytes and chamaephytes are among the most common life forms within the *Carex rupestris* cluster (Table 2).

### *Nardus stricta* cluster

This is a group of 51 species fairly differentiated in terms of their fit to the cluster pattern (the lowest S value was 0.12, 52% of the species had S value≥0.5, 17% had S value≥0.7). The species grouped here exhibit an interesting pattern of distribution with three major centres (Figure 2C): in the Eastern Fjords, in the central part of the northern coast (around Eyjafjörður), and in the western part of the country (with a clear hotspot in the Western Fjords). Boreo-temperate species are the most abundant (39%). Boreo-arctic montane species and arctic species account for 17% and 15%, respectively. Wide-boreal and boreal-montane species account for 12% each, 5% of the species belong to the temperate element (Figure 2C’). We recorded the presence of eight threatened species in this cluster. Hemicryptophytes and geophytes are the most common life forms (Table 2).

### *Saxifraga aizoides* cluster

This small cluster contains 25 species. The lowest S value was 0.13, 44% of the species had S value≥0.5, while 16% had an S value≥0.7. Species assigned to this cluster occur in the Eastern Fjords and neighbouring regions (Figure 2D). This can be regarded as the most localized cluster. There is no clear pattern in the biogeographical affinities of the cluster. Boreo-temperate species are most abundant (29%), arctic and boreal-montane and temperate species have 19% share each, while wide-boreal species account just for 5% of the total number of species in the cluster (Figure 2D’). The group comprises six threatened taxa. Hemicryptophytes are the most represented life forms in this cluster (Table 2).

### *Puccinellia maritima* cluster

**Figure 3:**
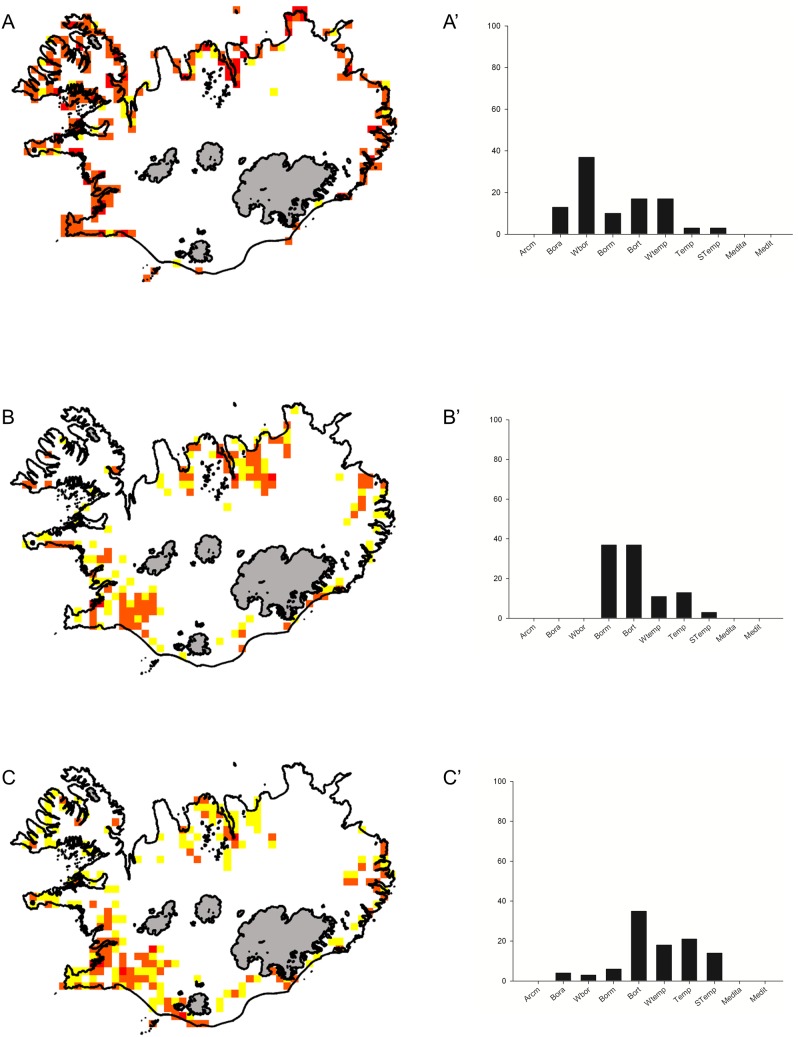
Distribution and biogeographical affinities of species clusters. Maps of species distribution in the clusters: *Puccinellia maritima* (A), *Potamogeton alpinus* (B), *Rumex longifolius* (C) (maps) and their biogeographic affinities (diagrams A’, B’, C’). For explanations see Figure 1.

This cluster species consists of 30 taxa. Their fit to the cluster pattern varies widely (the lowest S value was 0.10, 46% of the species had S value≥0.5 and 23% had S value≥0.7). Species from the cluster have a distinctly coastal distribution pattern (Figure 3A). The wide-boreal element is most represented (37%). Boreo-temperate and wide-temperate taxa account for 17% each, while boreo-arctic and boreal-montane species account for 13 and 10%, respectively (Figure 3A’). Temperate and southern-temperate taxa have a total share of 6%. The presence of six threatened species was recorded in the cluster. Hemicryptophytes, hydrophytes and therophytes are the most represented life forms (Table 2).

### *Potamogeton alpinus* cluster

Thirty-eight species were assigned to *Potamogeton alpinus* group. The lowest S value was 0.13, 44% of species had S value≥0.5, but only 16% had S value≥0.7. The distribution pattern here is rather complex. It seems that species from this cluster occur mainly in south-western part of Iceland, in the valleys of northern fjords: Skagafjörður and Eyjafjörður as well as in the areas south of Skjálfandi bay. Their occurrence in the eastern part of Iceland as well as along the southern coast is also apparent (Figure 3B). Boreal-montane and boreo-temperate species are most frequent in the cluster, accounting for 37% each. Wide-temperate, temperate and south temperate species account for 11, 13 and 3% respectively (Figure 3B’). The presence of seven threatened plant taxa was recorded. Hydrophytes are the most frequent life form (Table 2).

### *Rumex longifolius* cluster

The *Rumex longifolius* cluster is the largest one and consists of 80 species. The lowest S value recorded for the cluster is only 0.07, 42% of species have S≥0.5, while only 11% have S value≥0.7. The distribution pattern exhibited by species assigned to this group is not localised. The south-western part of the country seems to be the major area of their distribution. Taxa from the cluster occur, however, also around Eyjafjörður, along almost the whole southern coast and in the Eastern Fjords (Figure 3C). This pattern is broadly similar to the pattern present in the *Potamogeton alpinus* group, but the taxa in the *Rumex longifolius* cluster are more widespread. Boreo-temperate, temperate, wide-temperate and southern temperate species form the core of this cluster. More cold tolerant boreal taxa have a very small share, and the arctic element is completely absent (Figure 3C’). The *Rumex longifolius* cluster has the highest number of threatened taxa (Table 2). Hemicryptophytes and therophytes are the most common life forms in this group (Table 2).

### Factors shaping distribution patterns

We investigated bioclimatic and topographic factors in order to assess their importance in controlling the spatial distribution patterns present in Iceland.

**Figure 4:**
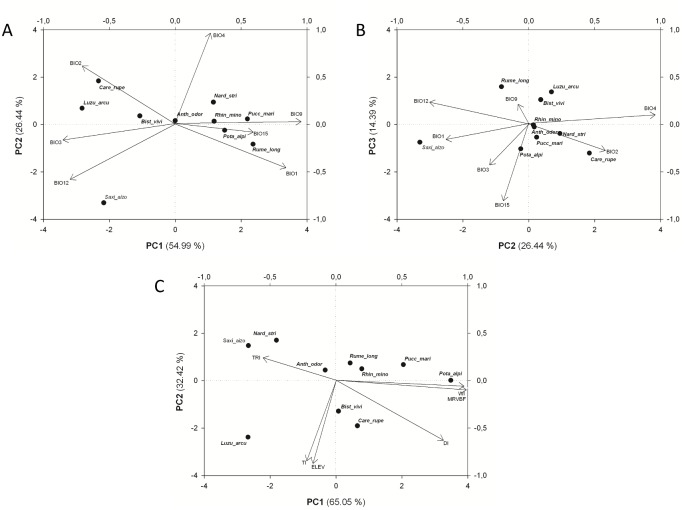
Environmental characteristics of the species clusters. Principal Component Analysis biplots: A. Bioclimatic variables PC2 vs. PC1, B. Bioclimatic variables PC3 vs. PC2, C. topographic variables PC2 vs. PC1.

In the case of the PCA carried out on the matrix containing information on the variation of bioclimatic variables, the first three principal components explained 95.82% of the total variance (Figure 4A, B). Three variables exhibited a high (>0.8) degree of correlation with the first principal component: mean temperature of the driest quarter (BIO9), annual mean temperature (BIO1) and isothermality (BIO3). The remaining variables showed a lower level of correlation with the first principal component (Table 3). Temperature seasonality (BIO4) and precipitation seasonality (BIO15) were highly correlated with the second and the third principal component, respectively (Table 3).

**Table 3:** Results of principal component analysis, correlations of variables and principal components.

Variable	PC1	PC2	PC3
Bioclimatic variables			
BIO1	**0.84**	−0.46	−0.16
BIO2	−0.71	0.61	−0.27
BIO3	−**0.85**	−0.16	−0.43
BIO4	0.27	**0.95**	0.10
BIO9	**0.96**	0.03	0.20
BIO12	−0.71	−0.57	0.24
BIO15	0.58	−0.08	−0.79
Topoclimatic variables			
ELEV	−0.47	−**0.88**	0.02
WI	**0.99**	−0.08	0.11
TI	−0.52	−**0.85**	0.08
DI	0.77	−0.63	−0.11
TRI	−**0.95**	0.24	0.19
MRVBF	**0.97**	−0.07	0.21

Correlations >0.8 are marked in bold face.

The *Carex rupestris*, *Luzula arcuata* and *Saxifraga aizoides* clusters were clearly separated from the rest of the analysed clusters along the first principal component (PC1) (Figure 4A). The second principal component separated *Saxifraga aizoides*, while values of PC2 coordinates for the remaining clusters were less differentiated (Figure 4A). The *Rumex longifolius* and *Puccinellia maritima* clusters exhibited the highest values of coordinates along the first principal component. The third principal component separated three clusters: *Rumex longifolius*, *Luzula arcuata* and *Bistorta vivipara* (Figure 4B).

In the case of the PCA carried out on the matrix containing information on the variation of topographic variables, the first two principal components explained 97.47% of the total variance (Figure 4C). Wetness index (WI), multiresolution index of valley bottom flatness (MRVBF) and terrain ruggedness index (TRI) were variables exhibiting a high degree of correlation with the first principal component, whereas elevation above sea level (ELEV) and total insolation (TI) showed the highest correlation with the second principal component (Table 3).

Only three species clusters *Potamogeton alpinus*, *Puccinellia maritima* and *Saxifraga aizoides* differed significantly in terms of both bedrock type and age when frequencies in the relevant classes were tested against the pooled data (Table 4). No significant differences were found concerning the bedrock pH (Table 4).

**Table 4:** Variation in categorical variables describing the bedrock type, its pH and age for each species cluster.

Variable andclass	Anth_odor	Bist_vivi	Care_rupe	Luzu_arcu	Nard_stri	Pota_alpi	Pucc_mari	Rhin_mino	Rume_long	Saxi_aizo	Pooled data
Lithology											
Lavas	20.66	26.39	25.40	18.57	14.77	**24.35**	**17.60**	20.99	25.46	**3.40**	23.30
Hyaloclastite	7.20	10.68	7.48	13.81	4.43	**4.71**	**1.11**	5.35	7.62	**1.62**	8.78
Extrusive rocks	68.66	60.20	64.83	66.53	78.42	**60.46**	**73.31**	68.29	59.70	**88.78**	64.36
Intrusions	0.40	0.35	0.11	0.50	0.25	**0.33**	**0.51**	0.36	0.36	**0.92**	0.37
Sands	3.08	2.39	2.18	0.60	2.13	**10.15**	**7.47**	5.00	6.86	**5.27**	3.20
pH											
Acidic	2.71	3.37	2.16	6.21	2.86	1.19	1.87	2.02	2.03	4.45	3.07
Basic orintermediate	97.29	96.63	97.84	93.79	97.14	98.81	98.13	97.98	97.97	95.55	96.93
Age											
HoloSedi	3.08	2.39	2.18	0.60	2.13	**10.15**	**7.47**	5.00	6.86	**5.27**	3.20
PostHist	1.25	1.68	0.70	1.63	0.86	**1.94**	**0.23**	1.12	1.29	**0.09**	1.44
PostPreHist	8.40	7.86	7.94	3.71	8.36	**11.44**	**8.80**	8.52	11.34	**1.13**	8.10
TertPleist	0.73	1.40	1.03	2.95	0.38	**0.16**	**0.09**	0.43	0.56	**0.09**	1.10
UppPleist	7.20	10.68	7.48	13.81	4.43	**4.71**	**1.11**	5.35	7.62	**1.62**	8.78
UppLow Pleist	26.28	35.43	41.33	33.79	12.26	**23.33**	**15.22**	23.71	24.85	**11.28**	29.96
Upp Terti	52.66	40.21	39.24	43.02	71.33	**47.93**	**66.58**	55.50	47.12	**79.58**	47.07
Ind	0.40	0.35	0.11	0.50	0.25	**0.33**	**0.51**	0.36	0.36	**0.92**	0.37

Values in bold are significantly different from those for the pooled data (p<0.05, χ^2^ test). Abbreviations for age classes: **HoloSedi**-holocene sediments; **PostHist**-postglacial, historic, younger than AD 871; **PostPreHist**-postglacial, prehistoric, older than AD 871; **TertPleist**-Tertiary and Pleistocene, older 11000 years; **UppPleist**-Upper Pleistocene, younger than 0.8 m.y.; **UppLowPleist**-Upper Pliocene and Lower Pleistocene, 0.8–3.3 m.y.; **UppTerti**-Upper Tertiary, older than 3.3 m.y.; **Ind**–Indefinite.

## Discussion

### Factors influencing the results of the analyses

Parameters describing exponents for weighing can be considered critical to how the spherical k-means clustering analysis performs [10]. We tested three different weighting schemes: W0.0, W0.5 and W1 (Figure S3). We noticed that main distribution patterns identified by the analysis remained fairly stable. The unweighted scheme (W0.0) produced one additional cluster of 21 species occurring mostly around Eyjafjörður. Moreover, in the analysis carried out using this scheme, the most widespread clusters were also among the largest in terms of assigned species. In case of less widespread clusters with localised pattern of occurrence, the W1 weighing scheme resulted in the highest species count per cluster, while the W0 scheme produced clusters with the lowest species count. For those reasons we decided to use W0.5 scheme as the most optimal (intermediate) both in terms of spatial patterns and the number of species per cluster. A similar strategy was employed by Preston et al. [11] in their analysis of spatial patterns in the British and Irish flora.

The question of choosing the right number of clusters has been investigated by different researchers. Their propositions involved different approaches from arbitrary formulas [35] to the methods based on Akaike information criterion [10]. These attempts showed that statistically optimal number of clusters is often less appropriate in terms of biogeographical significance, than the number of clusters chosen arbitrarily. Both Hill et al. [10] and Preston et al. [11] argue that arbitrary choice of the most meaningful and user-suitable number of clusters is better that acceptation of a statistically optimal number, especially in case of large datasets [10].

We analysed broad range of possible cluster numbers (k) ranging from k = 2 to k = 70. To assess the statistically optimal number of clusters we used the method developed by Hill et al. [10] and based on the Akaike information criterion. The results of this analysis showed that statistically optimal number of clusters was too high (438 species were divided into 45 clusters, ca.10 species per cluster) to provide a biogeographically meaningful overview. Similar results were obtained also by applying the elbow method that suggested that the optimal number of clusters should be 50 (data not shown). We would argue that in these circumstances there are strong arguments supporting arbitrary selection of the number of clusters, as the statistically optimal number of clusters is not meaningful in terms of biogeographical analysis. It should also be stressed that clustering techniques are very helpful in analysing patterns present in datasets, but they are unable to produce a single “correct” classification, as there are numerous ways of dividing up large datasets that are equally “correct”. All the methods designed to assist the researcher in the selection of the optimal number of clusters are aimed at finding a balance between maximum compression of the data (when all species are placed in one cluster) and maximum accuracy (when each species in assigned to its own cluster) but they do not provide a test of a model in the sense of testing a null hypothesis. Methods used to assess the results of clustering analyses give only a relative estimate of the information lost, and can not measure the quality of the model in an absolute sense, therefore their results are not conclusive in terms of the “correctness” of the model accepted.

The spherical k-means analysis conducted by us involved an allocation of the species into a predefined number of clusters. The goodness of fit was in our case quantified by the cosine measure S that is calculated for each species-cluster pair (Text S1). It is expected that some of the species will fit the cluster patterns well (i.e. they will exhibit high values of S). Some other species will be assigned to a cluster just because the value of S was the highest for a given species-cluster pair, although being generally low. Allocation of a species to a cluster in this case represents a “least bad” solution. Consequently, there are species in each cluster that may not perfectly follow the spatial pattern on the group. These species would usually produce “noise” that may potentially influence the results of other analyses (especially in case of the analysis of the environmental factors). In case of our study we included data from all the species assigned to the cluster, regardless the S value.

The spatial resolution applied by us needs also to be discussed. The collection of species occurrence data in Iceland can be described as “locality-based” in contrast to “grid-based” methodology that is used in some countries. It means that plant occurrence is not represented in the database just as a species-hectad pair, but is always accompanied by much more accurate location and relevant geographical coordinates. This means that the potential resolution of our analysis would be much more detailed than the 10×10 km hectad. Having this in mind we decided to employ topographic and climatic data with a resolution finer than 10×10 km. We would argue that using the same spatial resolution for clustering and the analysis of environmental variables would result in generalisation that could potentially blur relations between environmental variables and distribution pattern. We followed here the way of thinking presented by Whittaker et al. [36], who stated that some relationships between environmental variables and species distribution may be so fundamental that they operate across all scales without being easily detectable at all scales.

### Clusters and environmental variables

Our results showed that species clusters differed in terms of analysed environmental variables. Generally, temperature-related variables (BIO1, BIO3, BIO4 and BIO9) were the key factors responsible for differentiation between the clusters. Elevation and variables connected with the terrain sculpture (WI, TI, TRI and MRVBF) were among the most important topographic variables separating species clusters. The influence of the geological variables (related to the bedrock) was relatively weak, but significant in some cases.

It seems to us that analysed clusters can be divided into major groups reflecting their distribution and the characteristics derived from environmental variables. In case of climatic variables *Luzula arcuata* and *Carex rupestris* clusters can be considered as the most cold tolerant, while the remaining groups seem to prefer areas with moderate (e.g. *Anthoxanthum odoratum*) and mild climatic conditions (*Puccinellia maritima*, *Rumex longifolius*). This differentiation seems to be shaped mainly by the mean temperature.

There is, however, another interesting example of climatic differentiation present within the analysed dataset. This is the *Saxifraga aizoides* cluster, which is separated by a variable designed to quantify temperature seasonality (coefficient of variation calculated from monthly means). Values of this variable were the lowest in the case of *Saxifraga aizoides* group, suggesting a best fit to areas with the lowest temperature oscillations during the year.

It is clear that the relative frequency of arctic species followed also a temperature gradient: being the highest in the *Luzula arcuata*, *Carex rupestris* and *Bistorta vivipara* groups and intermediate or low in the remaining groups apart from *Puccinellia maritima*, *Potamogeton alpinus* and *Rumex longifolius*, where arctic species were not represented.

Topographical variables evidenced clear separation of the *Luzula arcuata* group. This cluster can be associated with high altitudes and heterogenous mountain landscape rich in ridges, tops and slopes. This topographic characteristic is well mirrored by harsh climatic conditions, as described above. Relatively high elevation, but remarkably lower topographic heterogeneity (when compared with the *Luzula arcuata* group) characterize two clusters: *Bistorta vivipara* and *Carex rupestris.* Two other groups, *Saxifraga aizoides* and *Nardus stricta*, seem to be best described by completely opposite characteristics: relatively low elevation and extremely high topographic heterogeneity (two topographic variables reach extreme values in the *Saxifraga aizoides* group: the highest value of TRI and the lowest value of MRVBF index). It seems, therefore, that climatic conditions are mainly responsible for the emergence of two different spatial patterns in these two groups. The results of PCA suggest high similarity of both clusters in the space of topographic variables, whereas in the space of climatic variables these two groups appear to be distant. Our results suggest that temperature seasonality may be responsible for this biogeographical differentiation.

Relatively mild climatic conditions and a flat and homogenous landscape seem to characterise the environments of two clusters: *Puccinellia maritima* and *Potamogeton alpinus*, dominated by coastal plants in case of the former and hydrophytes in case of the latter group. Interestingly, these two clusters differ significantly from the remaining clusters in terms of preferred bedrock (sand).

The results showed that the three most widespread clusters, *Bistorta vivipara*, *Anthoxanthum odoratum* and *Rhinanthus minor*, form a sequence of species with decreasingly ubiquitous ranges. On the PCA plot (analysis of bioclimatic variables, Fig. 4A) these three clusters form a clear gradient along PC1. PCA analysis carried out on topographic variables (Figure 4C) showed that elevation above sea level may also contribute to this differentiation. Generally, all three clusters were placed in the centre of PCA plots, suggesting that the species grouped here tend to occur in environments characterized by intermediate values of the investigated factors and hence explaining their wide distribution.

Temperature and precipitation are considered to be main factors shaping plant distribution patterns [37]. Our results support these findings and confirmed that in Iceland, temperature-related variables and elevation were the main factors responsible for differentiation between the main species clusters. In our previous study focused on the alien flora we showed that temperature and elevation were also among the main factors controlling the distribution of these species [24]. Surprisingly, however, the effect of precipitation-related variables was relatively weak in our analyses. Only precipitation seasonality (BIO15) was relatively highly correlated with the PC3, but the amount of the total variance explained by this principal component was low. It suggests that precipitation plays only a secondary role in shaping distribution patterns of the Icelandic flora.

Temperature and precipitation can be substantially modified locally by topographic factors such as elevation, aspect or slope effects [38]. Digital elevation models are currently among the most convenient sources of topographic data and are used to interpolate climatic data [32] as well as to calculate variables quantifying the potential amount of accessible resources that are essential to plant growth such as water, light, warmth and nutrients [39], [40]. Topographic data have also been successfully employed in biogeographical studies [41]–[43]. In our analyses topographic variables greatly contributed to the differentiation between clusters. Two indices TRI and MRVBF may serve as an excellent example here. Both variables were highly correlated with PC1 and separated clearly not only the *Luzula arcuata* cluster (containing cold tolerant mountain species), but also two clusters with a low frequency of arctic species: *Nardus stricta* and *Saxifraga aizoides*. These two clusters are rich in species belonging to more southern biome categories (including boreo-temperate and temperate species). It can be hypothesised that highly heterogeneous topography allow them to take advantage of the thick snow cover that accumulates in local depressions and sheltered locations. Our field observations suggest that many species from the *Nardus stricta* group inhabit local depressions and snowbeds. This pattern of local occurrence may protect plants from cold [44] by reducing temperature extremes and freeze-thaw cycles [45], [46], but also offers protection from wind damage, abrasion by ice crystals [47], winter desiccation and light damage (chlorophyll bleaching) [48], [49].

Geological factors (especially bedrock type) are among the most important factors shaping plant distribution patterns (e.g. [11], [50], [51]). The case of Iceland is, however, quite different. Our research evidenced no impact of the bedrock on differentiation between the major floristic clusters. This fact is a consequence of the uniform chemical composition of the bedrock. Geologists recognise three types of main extrusive rock types within the country [52], but chemical differences between them are minimal [53]. The *Puccinellia maritima* and *Potamogeton alpinus* clusters tend to prefer habitats shaped by sand as a predominant bedrock type. This is not surprising as both clusters are rich in hydrophytes and coastal plants. Members of *Saxifraga aizoides* cluster seems to be much less frequent on lavas, when compared with other floristic elements. However, this differentiation cannot be so easily explained. As discussed above, we suppose that other than geological factors play a key role in shaping this distribution pattern.

Earlier studies focused on factors controlling plant distribution in the arctic biome have showed that temperature plays a key role in shaping patterns of plant diversity both across the main biogeographical regions [15] and at the level of local flora [54]. It seems that Iceland may be in the future an excellent place to investigate the impact of climate change on plant distribution at high altitudes, due to the fact that distribution patterns are here controlled almost exclusively by climatic factors modified locally by terrain sculpture and altitude effects. Almost complete independence of distribution patterns from edaphic factors (such as bedrock characteristics), that are among main parameters influencing biogeographical patterns in other regions, is another special feature of Icelandic environment that make it suitable for analyses focused on the impact of climate change. Our study sets a baseline for such research.

### Protection of the Icelandic flora

The vast majority of the species mentioned in the Icelandic Red List of Plants [31] exhibit very scattered patterns of distribution that do not fit into any of the identified patterns particularly well. This is mirrored by their low cosine S values that are ≤0.5 in all cases. For that reason it would be difficult to point out cluster(s) of particular importance in terms of species protection. There are, however, some clusters with a high proportion of threatened species such as *Saxifraga aizoides* (32%), *Puccinellia maritima* (23%), *Carex rupestris* (23%) and *Rumex longifolius* (20%). The *Puccinellia maritima* cluster deserves special attention in terms of habitat protection. Threatened species in this cluster are mainly salt marsh species and can be treated also as an indicator of this rare habitat. The method employed by us did not, however, differentiate between salt marsh species and those much more frequent species typical of gravelly and rocky coasts.

Several aspects have to be taken into account when discussing protection of the Icelandic flora: protection aims, the presence of mechanisms and effectiveness. It seems that protection aims in Iceland should not be restricted only to clusters with the highest proportion of threatened species and should also facilitate the protection of unique patterns of species distribution. In other words conserving one rare species in a single place should go hand in hand with an attempt to protect distribution patterns present in the Icelandic flora. This question is particularly important in terms of climate change and its effects on plant distribution. It seems that spatial patterns of clusters dominated by arctic species (*Luzula arcuata*, *Carex rupestris*) are more threatened than those with significant proportions of boreal and temperate species. Callaghan et al. [54] stressed that arctic species will be most vulnerable to the climate change. They argued that the ecological amplitude of arctic taxa will narrow and their abundance will decrease during climate warming.

At present there is no match between the distribution of protected areas and distribution of the clusters with highest proportion of threatened species. Proposals were made in 2008 to establish several protected areas but they still did not pass the legislation process. It seems therefore that mechanisms are still not in place to ensure effective protection of the Icelandic flora.

## Conclusions

Our study allowed us to define and describe ten floristic elements in the native vascular flora of Iceland differing in terms of geographical distribution. We showed that climatic and topographic variables are mainly responsible for differentiation between the floristic elements defined by us. We did not record any significant impact of the bedrock type on the spatial patterns in the Icelandic flora.

## Supporting Information

Figure S1 **Akaike information criterion as a function of the number of clusters used in the SPHERIKM analysis.**
(PDF)

Figure S2 **Similarities between clusters identified at k = 10 and k = 45 (statistically optimal) expressed as a percent of shared species.**
(PDF)

Figure S3 **Result of using different weighting schemes in SHPERIKM.**
(PDF)

Text S1 **URLs to access datasets used during the present study.**
(PDF)

Text S2 **Cosine measure of similarity (S) for 20 most characteristic species from each cluster.**
(PDF)
