# Supplementary figures and images for: Distribution Patterns in the Native Vascular Flora of Iceland
Source: PLoS One. 2014 Jul 18;9(7):e102916. doi: 10.1371/journal.pone.0102916 (PMC4103864; doi:10.1371/journal.pone.0102916)

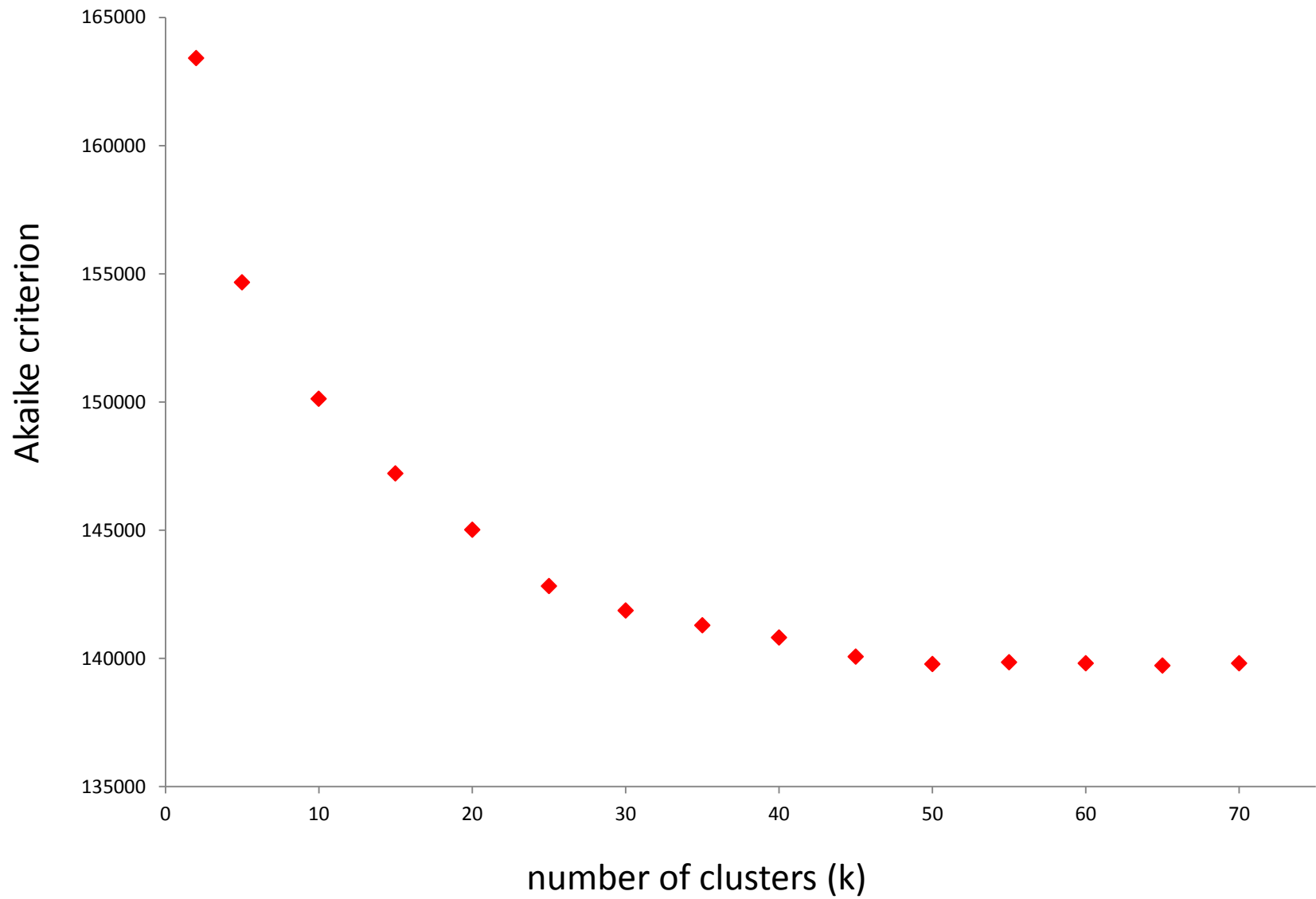

Supplement: Figure S1 — Akaike information criterion as a function of the number of clusters used in the SPHERIKM analysis. (PDF) [file pone.0102916.s001.pdf]

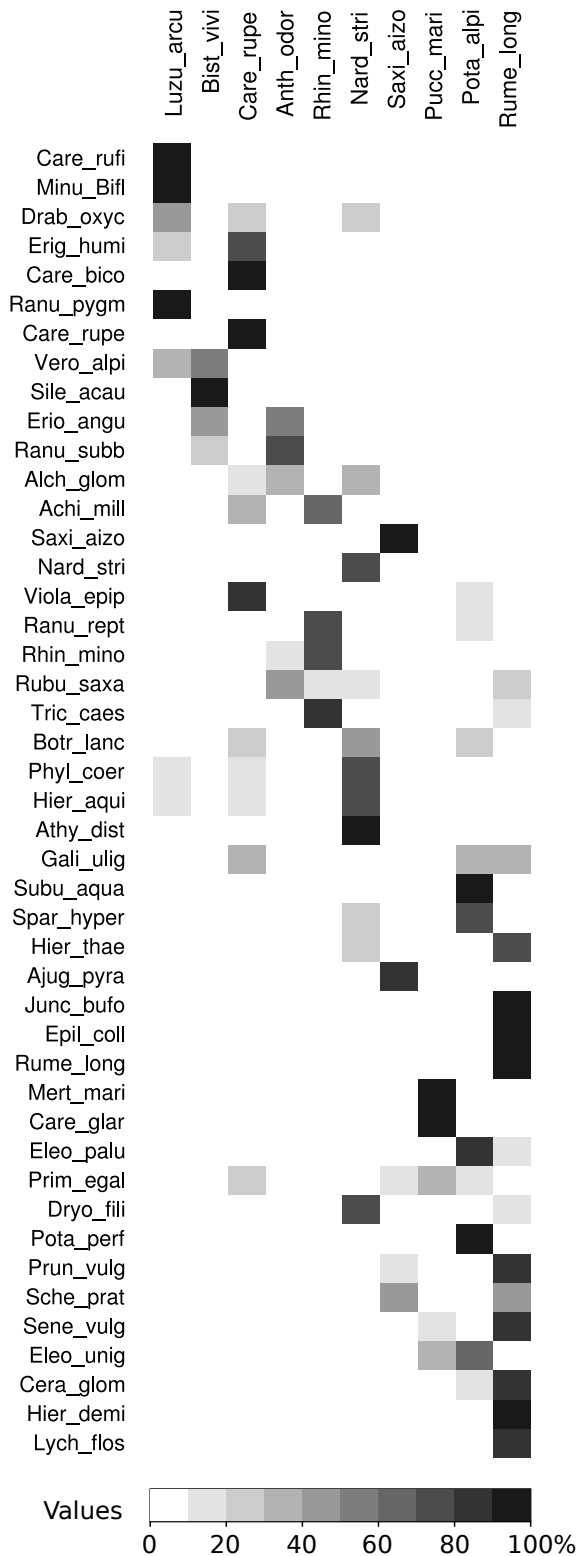

Supplement: Figure S2 — Similarities between clusters identified at k = 10 and k = 45 (statistically optimal) expressed as a percent of shared species. (PDF) [file pone.0102916.s002.pdf]
