# Supplementary material for: Distribution Patterns in the Native Vascular Flora of Iceland
Source: PLoS One. 2014 Jul 18;9(7):e102916. doi: 10.1371/journal.pone.0102916 (PMC4103864; doi:10.1371/journal.pone.0102916)

Exponents for weighting 0.000
Clusters 10
Randomizations 50
Shortlist factor 3.00
Quality relaxation factor 0.999000
Seed number reduction factor 0.80
Number of outer loops 10
Random start number 0

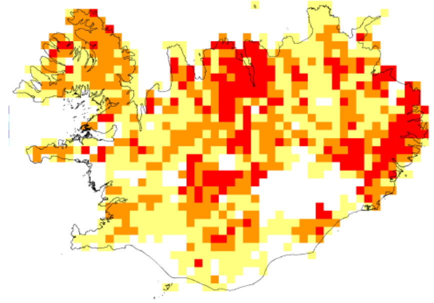

Luzula arcuata **25**

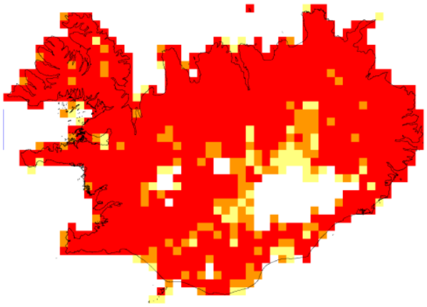

Empetrum nigrum **96**

X

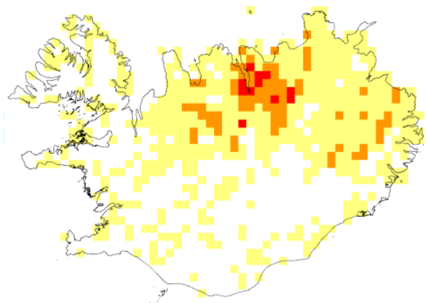

Carex rupestris **28**

Exponents for weighting 0.500
Clusters 10
Randomizations 50
Shortlist factor 3.00
Quality relaxation factor 0.999000
Seed number reduction factor 0.80
Number of outer loops 10
Random start number 0

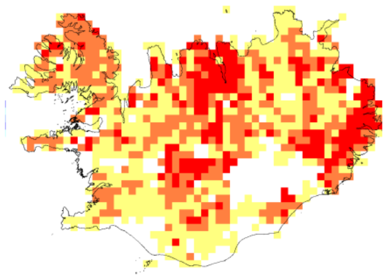

Luzula arcuata **25**

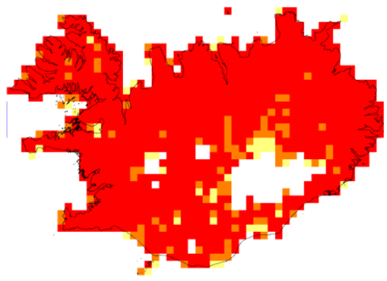

Bistorta vivipara **65**

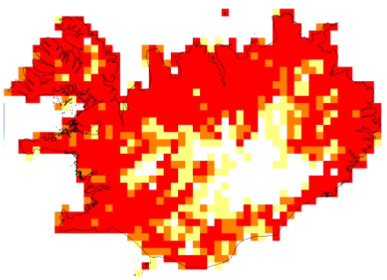

Anthoxanthum odoratum **53**

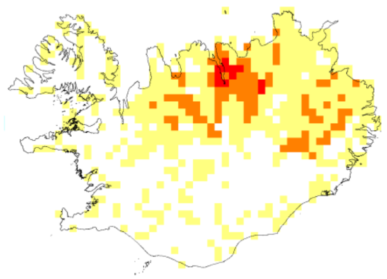

Carex rupestris **26**

Exponents for weighting 1.000
Clusters 10
Randomizations 50
Shortlist factor 3.00
Quality relaxation factor 0.999000
Seed number reduction factor 0.80
Number of outer loops 10
Random start number 0

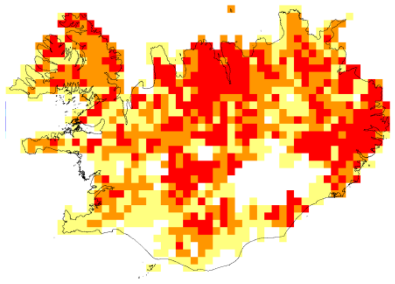

Omalotheca supina **31**

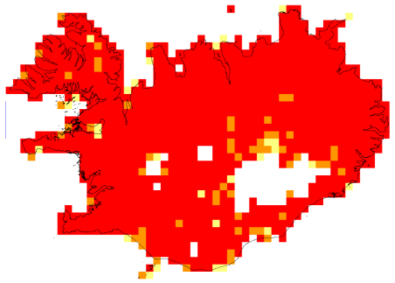

Silene acaulis **39**

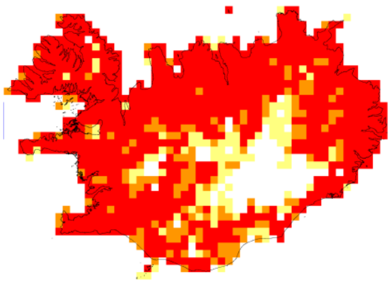

Ranunculus subborealis **57**

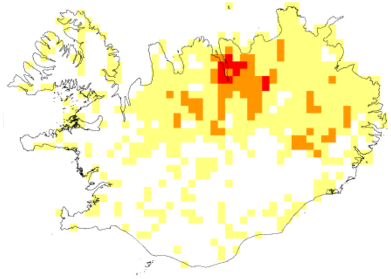

Carex rupestris **28**

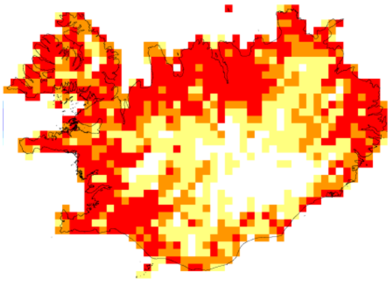

Rhinanthus minor **78**

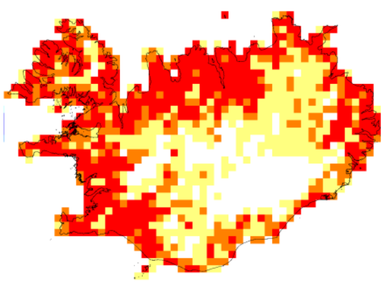

Rhinanthus minor **47**

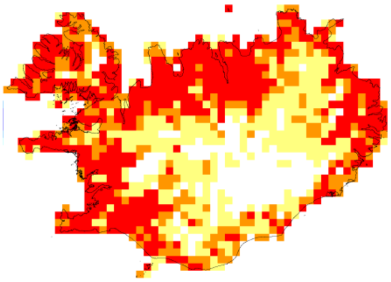

Rhinanthus minor **50**

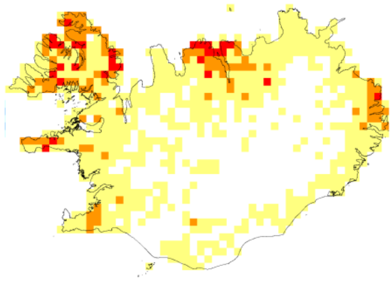

Gymnocarpium dryopteris **41**

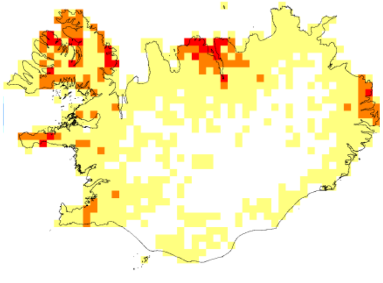

Nardus stricta **51**

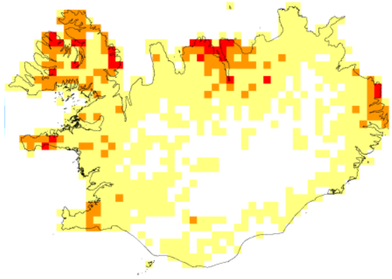

Nardus stricta **54**

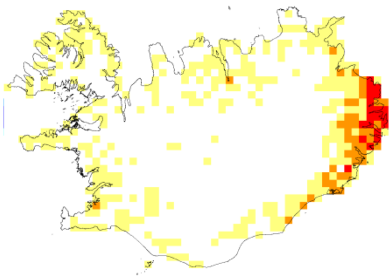

Trientalis europea **21**

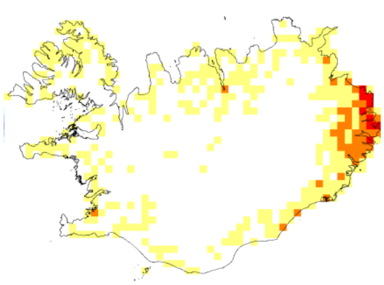

Saxifraga aizoides **25**

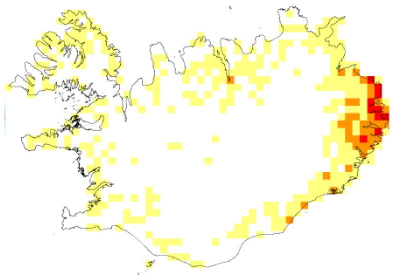

Saxifraga aizoides **23**

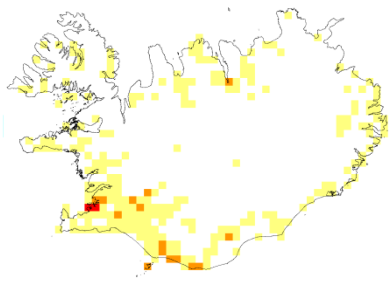

Cardamine hirsuta **37**

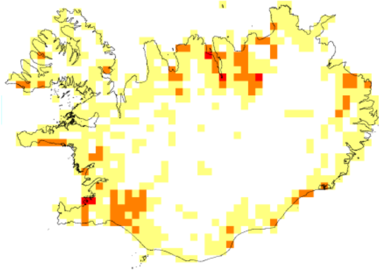

Potamogeton alpinus **38**

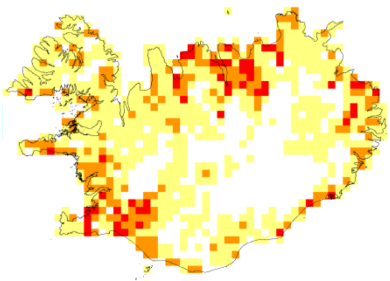

Ranunculus reptans **39**

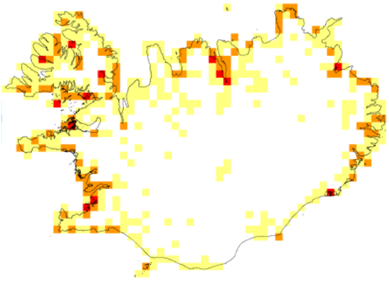

Puccinelia maritima **27**

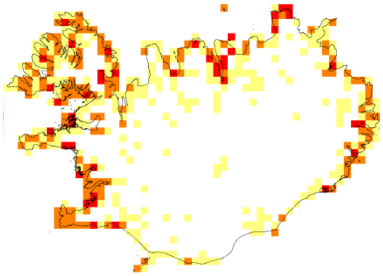

Puccinelia maritima **30**

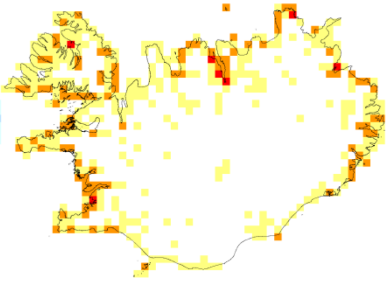

Puccinelia maritima **30**

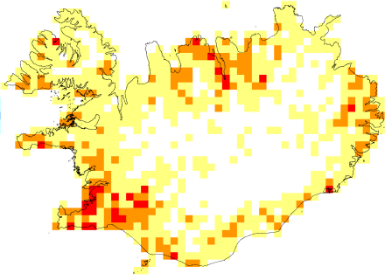

Rumex longifolius **70**

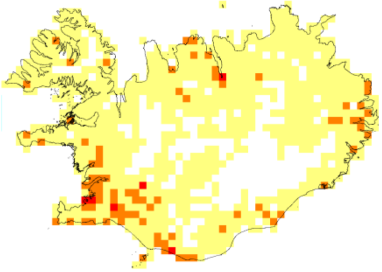

Rumex longifolius **80**

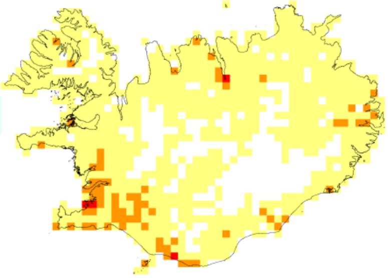

Rumex longifolius **89**

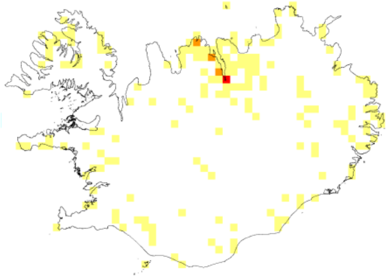

Primula egalikensis **21**

X

X

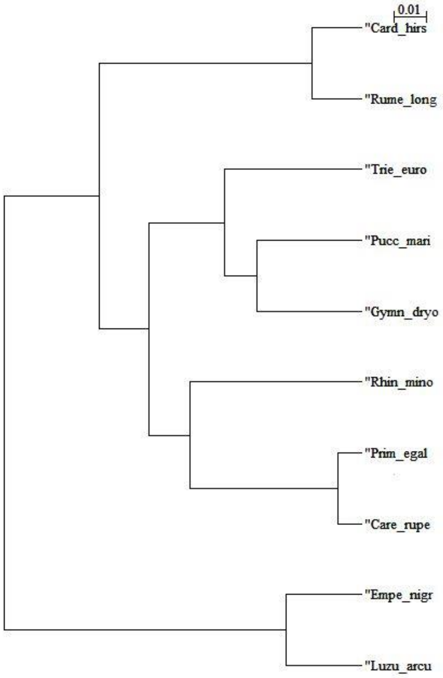

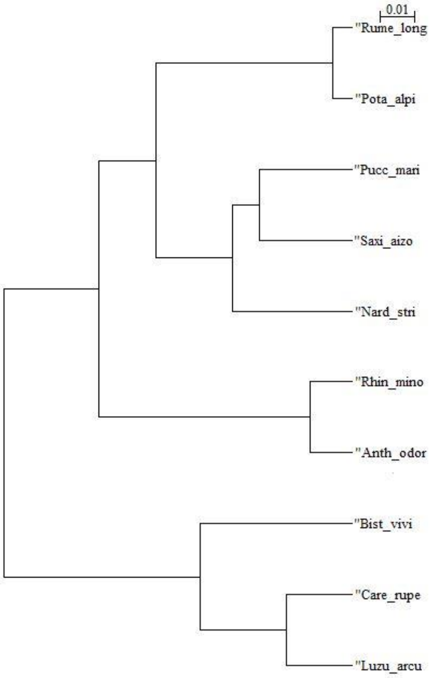

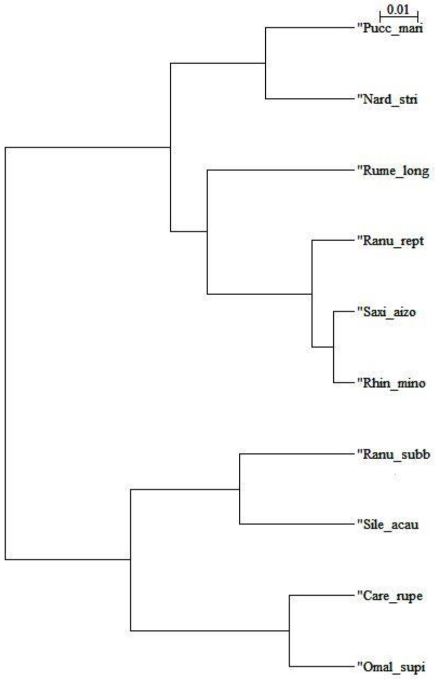

Supplement: Figure S3 — Result of using different weighting schemes in SHPERIKM. (PDF) [file pone.0102916.s003.pdf]
