# Supplementary material for: Distribution Patterns in the Native Vascular Flora of Iceland
Source: PLoS One. 2014 Jul 18;9(7):e102916. doi: 10.1371/journal.pone.0102916 (PMC4103864; doi:10.1371/journal.pone.0102916)
Supplement: Text S1 — URLs to access datasets used during the present study. (PDF) [file pone.0102916.s004.pdf]

Occurrence data used during this study were published in Global Biodiversity Information Facility (GBIF) by the Icelandic Institute of Natural History are available at the following URLs:

http://www.gbif.org/dataset/83447e40-f762-11e1-a439-00145eb45e9a

http://www.gbif.org/dataset/83435394-f762-11e1-a439-00145eb45e9a

http://www.gbif.org/dataset/83422c44-f762-11e1-a439-00145eb45e9a

Data can be also obtained upon request by contacting the corresponding author of the study Dr Pawel Wasowicz (pawel@ni.is).
