# Supplementary material for: Distribution Patterns in the Native Vascular Flora of Iceland
Source: PLoS One. 2014 Jul 18;9(7):e102916. doi: 10.1371/journal.pone.0102916 (PMC4103864; doi:10.1371/journal.pone.0102916)
Supplement: Text S2 — Cosine measure of similarity (S) for 20 most characteristic species from each cluster. (PDF) [file pone.0102916.s005.pdf]

Luzula arcuata cluster: *Luzula arcuata* 0.86, *Carex lachenalii* 0.86, *Epilobium anagallidifolium* 0.85, *Saxifraga rivularis* 0.84, *Arabis alpina* 0.84, *Saxifraga cernua* 0.81, *Ranunculus pygmaeus* 0.79, *Cerastium nigrescens* 0.78, *Sagina nivalis* 0.78, *Erigeron uniflorus* 0.75, *Minuartia biflora* 0.75, *Epilobium lactiflorum* 0.74, *Saxifraga tenuis* 0.71, *Ranunculus glacialis* 0.70, *Poa flexuosa* 0.70, *Pedicularis flammea* 0.70, *Phillipsia algida* 0.59, *Cardamine bellidifolia* 0.59, *Carex rufina* 0.58

Bistorta vivipara cluster: *Bistorta vivipara* 0.97, *Luzula spicata* 0.97, *Empetrum nigrum* 0.97, *Silene acaulis* 0.97, *Poa glauca* 0.97, *Cerastium alpinum* 0.97, *Salix herbacea* 0.97, *Equisetum arvense* 0.97, *Festuca rubra* 0.97, *Armeria maritima* 0.97, *Festuca vivipara* 0.97, *Carex bigelowii* 0.97, *Rumex acetosa* 0.97, *Galium normanii* 0.96, *Juncus trifidus* 0.96, *Thymus praecox* 0.96, *Thalictrum alpinum* 0.96, *Vaccinium uliginosum* 0.96, *Saxifraga caespitosa* 0.95, *Poa alpina* 0.95

Carex rupestris cluster: *Carex rupestris* 0.78, *Carex glacialis* 0.77, *Comastoma tenellum* 0.70, *Carex macloviana* 0.68, *Draba verna* 0.64, *Viola epipsila* 0.63, *Minuartia stricta* 0.62, *Draba nivalis* 0.62, *Lomatogonium rotatum* 0.62, *Carex krausei* 0.61, *Carex bicolor* 0.58, *Pyrola grandiflora* 0.48, *Antennaria alpina* 0.47, *Elymus alopex* 0.46, *Draba glabella* 0.44, *Carex nardina* 0.38, *Elymus kronokensis* 0.38, *Draba lactea* 0.37, *Campanula uniflora* 0.34, *Diapensia lapponica* 0.34

Anthoxanthum odoratum cluster: *Anthoxanthum odoratum* 0.95, *Alchemilla alpina* 0.94, *Luzula multiflora* 0.94, *Cerastium fontanum* 0.94, *Viola palustris* 0.94, *Carex nigra* 0.94, *Selaginella selaginoides* 0.94, *Equisetum pratense* 0.94, *Galium verum* 0.93, *Potentilla crantzii* 0.93, *Agrostis vinealis* 0.93, *Avenella flexuosa* 0.92, *Agrostis capillaris* 0.92, *Platanthera hyperborea* 0.92, *Carex capillaris* 0.92, *Juncus triglumis* 0.91, *Erigeron borealis* 0.91, *Carex rariflora* 0.91, *Comarum palustre* 0.90, *Equisetum palustre* 0.90

Rhinanthus minor cluster: *Rhinanthus minor* 0.91, *Leontodon autumnalis* 0.90, *Juncus alpinus* 0.90, *Poa annua* 0.89, *Epilobium palustre* 0.89, *Triglochin palustre* 0.89, *Stellaria media* 0.88, *Montia fontana* 0.87, *Menyanthes trifoliata* 0.85, *Viola canina* 0.85, *Caltha palustris* 0.85, *Carex lyngbyei* 0.83, *Carex canescens* 0.83, *Veronica serpyllifolia* 0.83, *Sagina nodosa* 0.82, *Pilosella islandica* 0.82, *Plantago maritima* 0.82, *Myosotis arvensis* 0.82, *Ranunculus reptans* 0.82, *Capsella bursa-pastoris* 0.82

Nardus stricta cluster: *Nardus stricta* 0.79, *Gymnocarpium dryopteris* 0.77, *Dactylorhiza maculata* 0.76, *Polystichum lonchitis* 0.76, *Diphasiastrum alpinum* 0.76, *Carex echinata* 0.75, *Listera cordata* 0.75, *Lycopodium annotinum* 0.74, *Omalotheca norvegica* 0.70, *Hieracium aquiliforme* 0.68, *Athyrium distentifolium* 0.66, *Hieracium lygistodon* 0.66, *Blechnum spicant* 0.61, *Papaver radicatum* 0.60, *Cornus suecica* 0.60, *Luzula sudetica* 0.60, *Sorbus aucuparia* 0.60, *Huperzia apressa* 0.60, *Hieracium alpinum* 0.59, *Carex paupercula* 0.57

Saxifraga aizoides cluster: *Saxifraga aizoides* 0.82, *Alchemilla faeroensis* 0.82, *Trientalis europaea* 0.80, *Campanula rotundifolia* 0.75, *Hieracium stoedvarense* 0.66, *Juncus castaneus* 0.64, *Hieracium anglicum* 0.63, *Hieracium acidotoides* 0.62, *Saxifraga paniculata* 0.56, *Saxifraga cotyledon* 0.56, *Ajuga pyramidalis* 0.50, *Hieracium strictophyllum* 0.49, *Carex pulicaris* 0.46, *Viola riviniana* 0.43, *Alchemilla glabra* 0.38, *Ranunculus auricomus* 0.34, *Oxalis acetosella* 0.34, *Juncus squarrosus* 0.34, *Vaccinium vitis-idea* 0.27, *Populus tremula* 0.26

Puccinellia maritima cluster: *Puccinellia maritima* 0.81, *Mertensia maritima* 0.78, *Carex glareosa* 0.78, *Honckenya peploides* 0.78, *Puccinellia capillaris* 0.73, *Atriplex glabriuscula* 0.73, *Stellaria humifusa* 0.70, *Cochlearia officinalis* 0.69, *Carex mackenziei* 0.68, *Cakile maritima* 0.66, *Carex subspathacea* 0.61, *Gentianopsis detonsa* 0.61, *Carex ramenskii* 0.56, *Triglochin maritima* 0.51, *Ligusticum scoticum* 0.47, *Sagina subulata* 0.44, *Argentina egedii* 0.44, *Galium trifidum* 0.43, *Zostera angustifolia* 0.41, *Urtica urens* 0.37

Potamogeton alpinus cluster: *Potamogeton alpinus* 0.77, *Potamogeton gramineus* 0.77, *Eleocharis palustris* 0.77, *Myriophyllum alternifolium* 0.74, *Carex limosa* 0.73, *Sparganium hyperboreum* 0.72, *Veronica scutellata* 0.68, *Potamogeton berchtoldii* 0.65, *Potamogeton perfoliatus* 0.65, *Subularia aquatica* 0.62, *Eleocharis acicularis* 0.60, *Eleocharis uniglumis* 0.60, *Limosella aquatica* 0.59, *Carex viridula* 0.58, *Juncus bulbosus* 0.58, *Rorippa islandica* 0.56, *Oxycoccus microcarpus* 0.52, *Utricularia minor* 0.50, *Potamogeton praelongus* 0.48, *Potamogeton natans* 0.47

Rumex longifolius cluster: *Rumex longifolius* 0.76, *Prunella vulgaris* 0.74, *Juncus bufonius* 0.73, *Angelica sylvestris* 0.72, *Poa trivialis* 0.72, *Elytrigia repens* 0.72, *Vicia cracca* 0.72, *Filipendula ulmaria* 0.71, *Alopecurus geniculatus* 0.71, *Ranunculus repens* 0.70, *Epilobium collinum* 0.69, *Tripleurospermum maritimum* 0.68, *Veronica officinalis* 0.68, *Carum carvi* 0.67, *Senecio vulgaris* 0.66, *Plantago major* 0.65, *Hieracium thaectolepium* 0.64, *Callitriche palustris* 0.64, *Fragaria vesca* 0.64, *Hieracium holopleurum* 0.63
